# Engineering aligned human cardiac muscle using developmentally inspired fibronectin micropatterns

**DOI:** 10.1038/s41598-021-87550-y

**Published:** 2021-06-01

**Authors:** Ivan Batalov, Quentin Jallerat, Sean Kim, Jacqueline Bliley, Adam W. Feinberg

**Affiliations:** 1 Department of Materials Science and Engineering, Carnegie Mellon University, 5000 Forbes Avenue, Pittsburgh, Pennsylvania 15213 USA; 2 Department of Biomedical Engineering, Carnegie Mellon University, 5000 Forbes Avenue, Pittsburgh, Pennsylvania 15213 USA

**Keywords:** Biomedical engineering, Bioinspired materials, Tissue engineering, Cardiovascular biology

## Abstract

Cardiac two-dimensional tissues were engineered using biomimetic micropatterns based on the fibronectin-rich extracellular matrix (ECM) of the embryonic heart. The goal of this developmentally-inspired, in vitro approach was to identify cell–cell and cell-ECM interactions in the microenvironment of the early 4-chambered vertebrate heart that drive cardiomyocyte organization and alignment. To test this, biomimetic micropatterns based on confocal imaging of fibronectin in embryonic chick myocardium were created and compared to control micropatterns designed with 2 or 20 µm wide fibronectin lines. Results show that embryonic chick cardiomyocytes have a unique density-dependent alignment on the biomimetic micropattern that is mediated in part by N-cadherin, suggesting that both cell–cell and cell-ECM interactions play an important role in the formation of aligned myocardium. Human induced pluripotent stem cell-derived cardiomyocytes also showed density-dependent alignment on the biomimetic micropattern but were overall less well organized. Interestingly, the addition of human adult cardiac fibroblasts and conditioning with T3 hormone were both shown to increase human cardiomyocyte alignment. In total, these results show that cardiomyocyte maturation state, cardiomyocyte-cardiomyocyte and cardiomyocyte-fibroblast interactions, and cardiomyocyte-ECM interactions can all play a role when engineering anisotropic cardiac tissues in vitro and provides insight as to how these factors may influence cardiogenesis in vivo.

## Introduction

Understanding how cardiomyocytes organize into an aligned heart muscle tissue is critically important to a wide range of applications from in vitro disease modelling^1–3^ to in vivo heart repair^4–6^. In the adult human heart, the myocardium is organized in a complex anisotropic structure with layers of aligned cardiomyocytes (heart muscle cells) wrapped in different orientations in order to support its pumping ability. This complex structure is formed during embryonic morphogenesis, where the heart starts as a linear tube and transforms into its 4-chambered structure. Though significant research has been performed to understand how cardiomyocytes organize into an aligned tissue^7–10^, the individual physical, mechanical and biochemical factors that drive this cardiomyocyte alignment are largely unknown. Here we sought to gain insight into how cell–cell and cell-matrix interactions potentially guide cardiomyocyte organization by using a 2D engineered in vitro system.

It is clear that the developing heart contains specific structural, mechanical and/or biochemical cues critical to cardiomyocyte organization; however, it is difficult to observe the specific cell–cell and cell matrix interactions in the mammalian heart in utero. Alternatively, cardiomyocyte organization has been investigated in vitro with engineered substrate topography^11–15^, applied mechanical forces^10,16–18^, 3D microstructure^19–22^, substrate stiffness^23–28^, and different extracellular matrix (ECM) compositions^29^. For example, Bian et al. utilized structural cues to align cardiomyocytes around hexagonal posts to replicate the anisotropic nature of the myocardium and found increased electrical and structural maturation of these cells^30^. Gao et al used multiphoton 3D printing to create grid-like patterns to simulate the structure of native ECM fibers within the heart^31^. Micropatterns have also proven to be effective in guiding cardiomyocyte alignment where researchers have used components of the ECM found in the embryonic heart^32^. Specifically, fibronectin has widely been used to engineer 2D monolayers of cardiac muscle tissue using both primary and stem cell derived cardiomyocytes^7,8,33–35^. However, this previous work has typically used simple geometric line patterns, such as 20 µm wide, 20 µm spaced (20 × 20) fibronectin line patterns^7^. In contrast, the fibronectin-rich ECM in the embryonic myocardium has vastly different fiber dimensions and overall structure, raising the question as to whether these micropatterns have biological significance.

Here we have implemented a developmentally-inspired approach to understand the microenvironmental factors that guide in vitro cardiac tissue formation, specifically how cell–cell and cell–matrix adhesions interact during this process in the context of 2D ECM. To address this directly, we used the chick embryo as a tractable model system to study early cardiogenesis in a 4-chambered vertebrate heart. Specifically, we imaged fibronectin in the left ventricle of embryonic chick hearts and used this to generate a biomimetic micropattern that mimics the native fibrillar ECM, and compared this to standard line patterns as controls. Primary chick cardiomyocytes and human induced pluripotent stem cell-derived cardiomyocytes were used to engineer 2D myocardium in order to evaluate how the developmental state, cellular composition and species influences tissue formation. Using cell alignment as an overarching metric of anisotropic tissue formation, our goal was to start to decouple the role of cell type, cell density, matrix patterning, matrix assembly and developmental state. Our results provide insight into how cell–cell and cell-matrix interactions work in concert with the fibronectin micropattern to influence the organization of cardiac muscle tissue in vitro.

## Results

### Bioinspired fibronectin micropatterns from the embryonic heart

**Figure 1:**
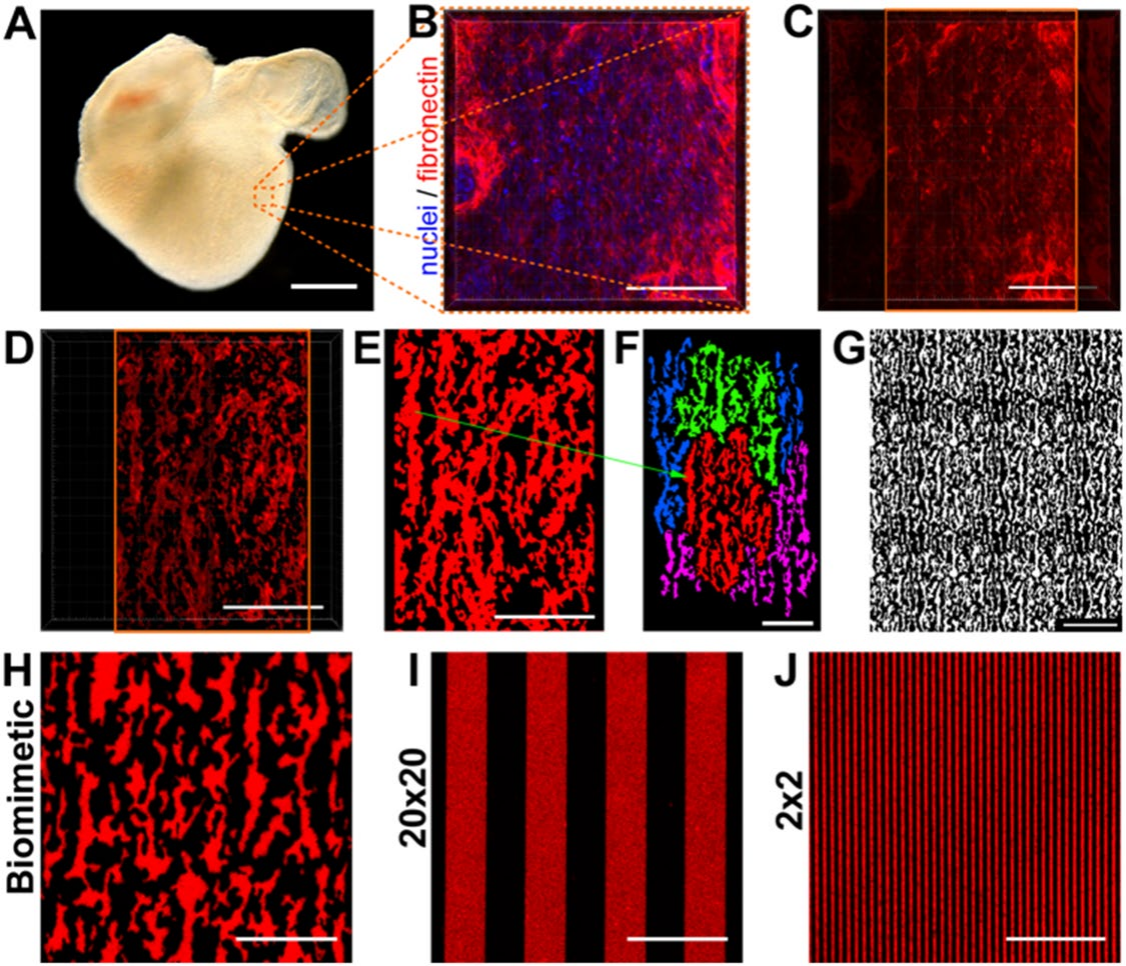
Approach to engineer a biomimetic 2D micropattern based on fibronectin ECM in the left ventricle of the embryonic chick heart. (**A**) Darkfield image of a 6-day-old embryonic chick heart. (**B**) Representative confocal 3D image (top down) of embryonic chick myocardium stained for nuclei (blue) and fibronectin (red). (**C**) The region of image in (**B**) used for the pattern generation, removing the nuclei and the high-intensity fibronectin areas on the edges corresponding to forming blood vessels. (**D**) Image in (**C**) processed to remove any features less than 1 µm in length, which are below the resolution of the photomask to be made. (**E**) Max intensity projection of the fibronectin ECM in (**D**) and conversion to a binary image. (**F**) The biomimetic pattern unit cell used in this study, composed of parts of four separate images combined into a single image, where each color represents the features from a specific image. The red region corresponds to the red pattern in (**E**), with the green arrow showing an example of a transferred feature. (**G**) The biomimetic unit cell in (**F**) is arrayed in 2D to create large area patterns in the photomask. (**H**) Representative image of the biomimetic micropattern microcontact printed using fluorescently-labeled fibronectin on PDMS-coated coverslips, confirming the fidelity of the protein transfer. (**I** and **J**) Representative images of the control micropatterns consisting of fibronectin (**I**) 20 × 20 and (**J**) 2 × 2 line patterns. Scale bars are (**A**) 500 µm, (**B-F**) 50 µm, (**G**) 100 µm, and (**H-J**) 50 µm. GIMP software was used to generate and edit the pattern (https://www.gimp.org/downloads/.).

**Figure 2:**
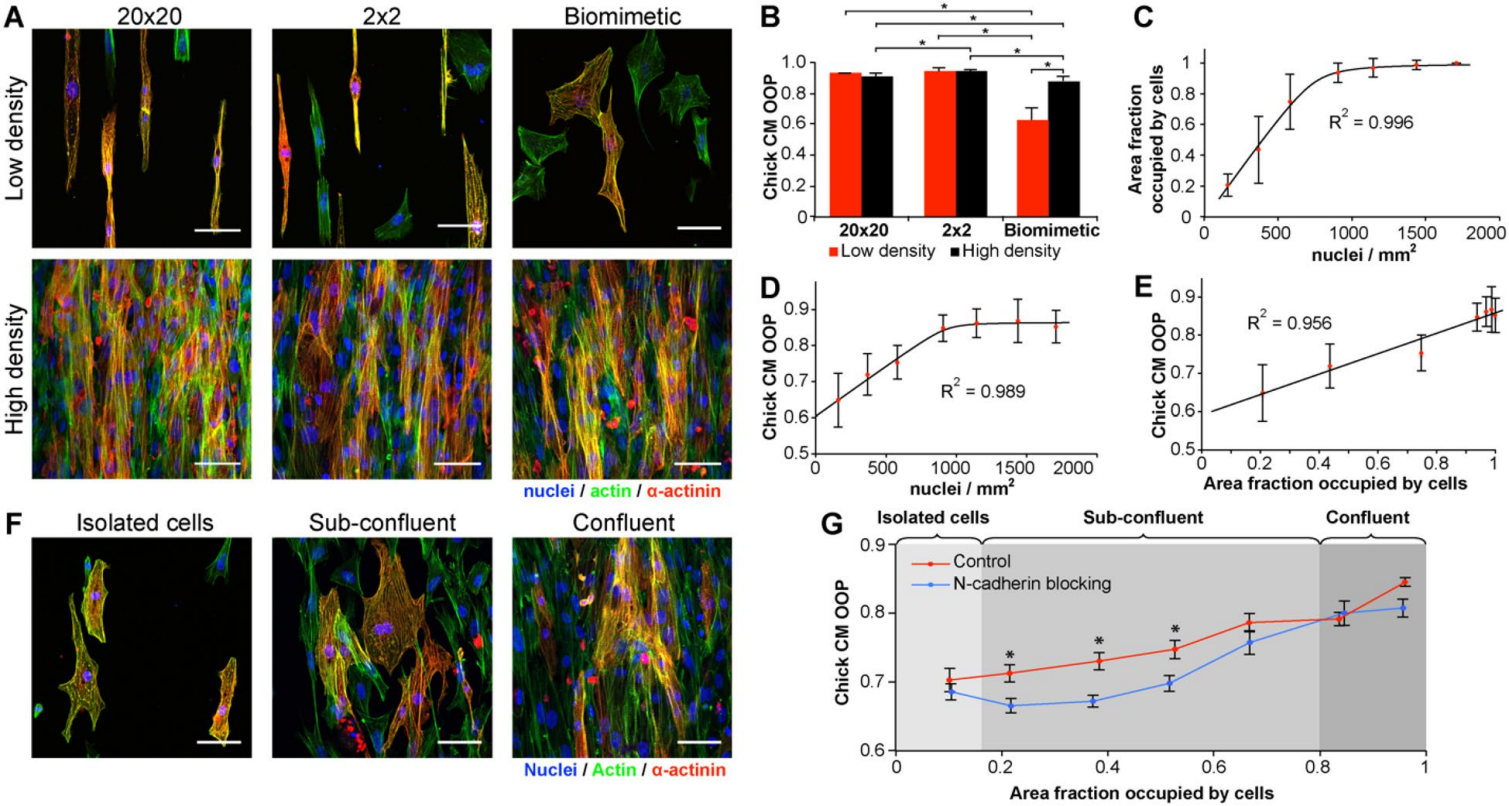
Analysis of enriched embryonic chick cardiomyocytes cultured on the fibronectin micropatterns at various cell densities. (**A**) Representative images showing pattern-dependent differences in cell morphology at low density (isolated cells) and high density (monolayer). (**B**) Chick cardiomyocyte OOP analysis at low and high cell densities (n ≥ 15) showing that only the biomimetic micropattern shows a density-dependent difference in cardiomyocyte alignment. At high density, cardiomyocyte alignment is significantly different between micropatterns, but still achieves a comparable OOP value of ~0.9. Two-way ANOVA with Tukey’s posthoc, * indicates p<0.05, n ≥ 15. (**C**) On the biomimetic micropattern, the area fraction occupied by cells increases linearly with cell density up until ~1000 nuclei/mm^2^, which corresponds to when the cells reach 100% confluence. (**D**) On the biomimetic micropattern, chick cardiomyocyte OOP also increases linearly with cell density up until ~1,000 nuclei/mm^2^, leveling off after confluence is reached. (**E**) Chick cardiomyocyte OOP on the biomimetic micropattern as a function of surface coverage showing a continuous, linear increase in cell alignment until confluence is reached. (**F**) Chick cardiomyocytes seeded at low, intermediate, and high densities on the biomimetic pattern produce isolated, sub-confluent and confluent cells, respectively. (**G**) Plot of chick cardiomyocyte OOP as a function of substrate coverage with and without N-cadherin blocking antibodies. Shaded regions indicate isolated cells that never contact one another, sub-confluent cells that contact one another, and confluents cells that are in continuous cell–cell contact. At intermediate cell densities ranging from 0.2 to 0.6 fractional substrate coverage, cells cultured with N-cadherin blocking antibodies had significantly lower OOP compared to controls, (n ≥ 18), *indicates p < 0.05 based on two-way ANOVA with Tukey post hoc performed on OOP values split into groups based on the cell density. Scale bars are 50 µm. Error bars in (**B-E**) are standard deviation and in (**G**) are standard error.

To study how a developmentally-inspired matrix influences cardiac tissue formation, we first engineered an ECM protein micropattern that mimics the structure of fibronectin in the 6-day-old embryonic heart. This timepoint was selected because our previous work has shown that at this timepoint aligned myocardium has formed with a fibronectin-rich ECM and very few capillaries^36^. To do this, we isolated embryonic chick hearts (Fig. 1A), and then fixed, fluorescently stained, and imaged the fibronectin ECM in the wall of the left ventricle (Fig. 1B). At this stage of development, the majority of the myocardium is trabeculated, with only a few layers of compacted and aligned cardiomyocytes in the ventricular wall. Images of compacted, ventricular myocardium were cropped to remove fibronectin corresponding to forming blood vessels or the adjacent epicardium (Fig. 1C). The image was then filtered to remove features < 1 µm in size (Fig. 1D), which is the resolution limit of the manufactured photomask. The filtered image was then converted into a 2D binary image (Fig. 1E) with dimensions approximating that of the original fluorescent fibronectin image. Finally, regions from multiple 2D binary images were combined together to construct a “unit cell” of the biomimetic pattern (Fig. 1F) that could then be arrayed in 2D to create a photomask that covers a 5 × 5 mm area (Fig. 1G). Established microcontact printing methods were then used to generate PDMS elastomeric stamps and transfer fibronectin to the surface of PDMS-coated coverslips (Supplementary Fig. S1). The fidelity of protein transfer during microcontact printing was validated using fluorescently-labeled fibronectin for the biomimetic (Fig. 1H), 20 × 20 (Fig. 1I) and 2 × 2 (Fig. 1J) micropatterns.

### Density-dependent alignment of chick cardiac cells on biomimetic fibronectin micropatterns

To evaluate cell response to the fibronectin micropatterns, we first used embryonic chick cardiomyocytes from 8-day-old embryos because these primary cells are from a developmental time point similar to the biomimetic pattern we generated, and the slightly older and larger hearts enabled us to isolate substantially more cells than 6-day-old hearts. We seeded the cardiomyocytes at low density (60,000 cells/cm^2^) and high density (450,000 cells/cm^2^). The low density was chosen to achieve a culture of isolated cells that were dominated by cell-matrix contact and had minimal cell–cell contact. In contrast, the high density was chosen to generate a continuous, confluent monolayer that maximized cell–cell contact. After 3 days of culture, cells were fixed, stained for nuclei, actin, and sarcomeric α-actinin, and imaged using confocal microscopy. Culture duration was chosen based on the cardiomyocyte’s ability to spread on the micropatterns and to limit the proliferation of non-cardiomyocytes (mainly fibroblasts). In general, cardiomyocytes appeared rounded (day 1 post-seeding) and had not fully spread on the patterns until day 3. By day 5 post seeding, non-cardiomyocytes (present in the chick heart isolation) demonstrated higher levels of proliferation that would skew cardiomyocyte alignment analysis.

The morphology and alignment of chick cardiomyocytes on the biomimetic pattern showed a unique density dependence that was not observed on the 20 × 20 and 2 × 2 control patterns. Qualitatively, cardiomyocytes were well aligned on the 20 × 20 and 2 × 2 control patterns with an elongated morphology in the pattern direction, and with no clear difference between high and low density (Fig. 2A). In contrast, cardiomyocytes at low density on the biomimetic pattern were more spread out and not as well aligned as on the controls. Indeed, though cell area did not appear to be significantly different between chick cardiomyocytes cultured at low density on the different patterns (BM, 2 × 2, and 20 × 20), an increase in cell aspect ratio was observed in both the 20 × 20 and 2 × 2 patterns when compared to biomimetic pattern (Supplementary Fig. S2). At high density, cardiomyocyte alignment on the biomimetic pattern increased and was similar to the control patterns. To quantify this response, we used a custom MATLAB script to measure the alignment of actin filaments by calculating the Orientational Order Parameter (OOP), which ranges from 0 for a perfectly isotropic distribution of myofibrils to 1 for perfectly anisotropic (parallel) myofibrils^7^. Since actin is organized parallel to the direction of sarcomere contraction within cardiomyocytes, the OOP also serves as an indirect measure of sarcomere alignment. However, additional features, like measurements of sarcomere spacing, were not included in this study. Chick cardiomyocyte OOP on the biomimetic pattern was significantly higher for the high-density seeding than for the low density (Fig. 2B). This is in distinct contrast to the 20 × 20 and 2 × 2 control patterns, where chick cardiomyocyte alignment was high and independent of seeding density.

To better understand the effect of cell density on the biomimetic pattern-driven tissue alignment, we seeded chick cardiomyocytes over a range of densities from 60,000 cells/cm^2^ to 450,000 cells/cm^2^. As expected, the fraction of surface area occupied by cells increased linearly with cell density, up until the cells reached confluence (Fig. 2C). Given that alignment on the biomimetic pattern was low at low cell densities, it was unexpected that chick cardiomyocyte OOP increased linearly with cell density (Fig. 2D). Importantly, by plotting cardiomyocyte OOP as a function of the surface area covered by cells, we found that the increase in alignment was linear with surface coverage (Fig. 2E, F). This strongly suggests that the density-dependent alignment on the biomimetic pattern was due at least in part to an increase in cell–cell interactions that occur more frequently with higher cell surface coverage. It appears that as the cardiomyocytes begin to directly touch one another, these direct cell–cell contacts work in concert with the underlying biomimetic micropattern to increase alignment.

We hypothesized that cell–cell interactions were indeed involved in the density-dependent alignment on the biomimetic pattern, so we next sought to systematically perturb these interactions. There are a number of potential cell–cell adhesion and gap junction proteins involved during direct cardiomyocyte coupling including cadherins and connexins^37–39^. To test this we focused on N-cadherin, a trans-membrane adhesion protein that is one of the most abundant cell–cell junctions between cardiomyocytes and important for differentiation and myofibrillogenesis^40,41^. Importantly, N-cadherin junctions serve as attachment sites for actin filaments and therefore can directly affect actin alignment^42^. To inhibit N-cadherin, we used blocking antibodies as previous studies have shown that introducing blocking antibodies reduces the amount of N-cadherin junctions^43,44^. To determine the optimal antibody concentration, we cultured cardiomyocytes with various concentrations of the blocking antibodies and looked at N-cadherin localization in cells (Supplementary Fig. S3). The results showed that without the blocking antibodies N-cadherin is primarily expressed on the cell membrane along cell–cell interfaces. With increased blocking antibody concentration, N-cadherin becomes internalized indicating that it is no longer involved in the formation of cell–cell junctions. The antibody concentration of 15.2 µg/mL was chosen to analyze the effect of blocking on cardiomyocyte alignment as almost no N-cadherin along cell–cell interface was observed at this concentration. We incubated cardiomyocytes on the biomimetic pattern at various cell densities ranging from 60,000 cells/cm^2^ to 450,000 cells/cm^2^ with and without N-cadherin antibodies for 3 days and measured their alignment along with the surface average. The results showed that the effect of N-cadherin blocking is dependent on cell density (Fig. 2G). At the lowest cell density blocking has no effect on alignment as cell–cell contacts are rare. With increased cell density, the cardiomyocyte OOP in the control group starts increasing, while the OOP of cells with blocking antibodies remains the same. This suggests that N-cadherin-based cell–cell junctions do influence cell alignment and are responsible for the observed increase in cardiomyocyte OOP. Finally, at high cell densities approaching a confluent monolayer, OOP of cells with blocking antibodies increases and converges with the OOP of the control group. We attribute this to the increased influence of the other types of interaction that we did not inhibit, which eventually renders N-cadherin blocking ineffective.

Another important factor that may influence the alignment of cardiac monolayers is heterogeneity in the cardiomyocyte population. Although the cardiomyocyte harvesting protocol includes purification via pre-plating, the final cell population still contains a small number of non-cardiomyocytes, primarily consisting of cardiac fibroblasts. These fibroblasts proliferate over time, lowering cardiomyocyte purity and potentially influencing OOP. Using sarcomeric α-actinin staining, we were able to detect cardiomyocytes and fibroblasts in cardiac tissues and independently analyze the alignment of each cell type. We found that while non-cardiomyocyte alignment in a monolayer matches that of cardiomyocytes, non-cardiomyocyte alignment in low-density culture was significantly reduced compared to the cardiomyocyte OOP (Supplementary Fig. S4). Further, we engineered fibroblast-rich cell monolayers on fibronectin patterns and found that they have lower alignment compared to cardiac tissues on all patterns (Supplementary Fig. S4). These findings suggest that non-cardiomyocytes, primarily cardiac embryonic fibroblasts, have an overall lower alignment on fibronectin patterns. This is important because it indicates that cell type, and potentially also interactions between different cell types, can decrease cardiomyocyte OOP in high density monolayers. However, this conclusion cannot be fully verified without obtaining a 100% pure population of chick embryonic cardiomyocytes, which is challenging using primary cells.

### Biomimetic fibronectin micropatterns influence cardiomyocyte adhesion and alignment

**Figure 3:**
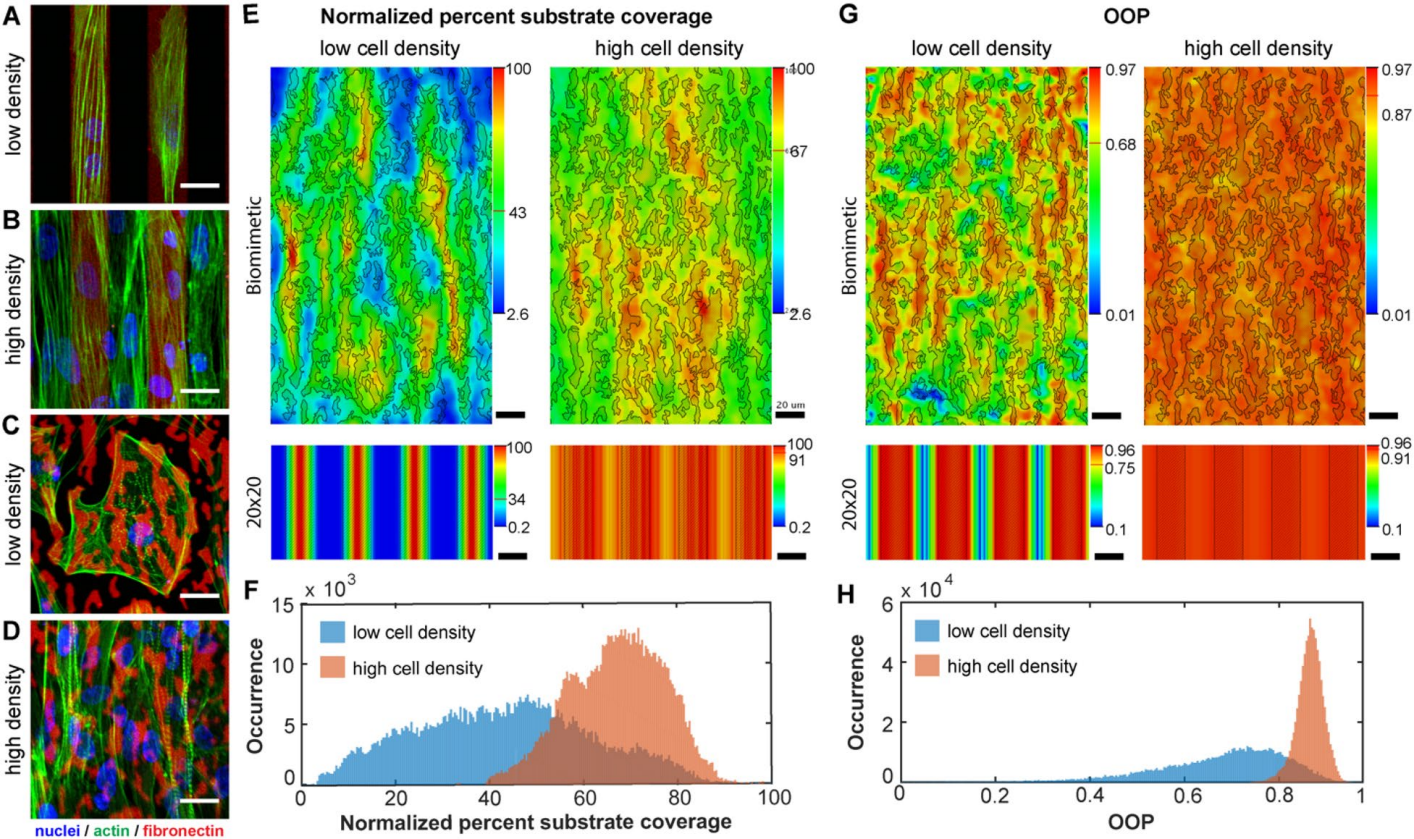
Analysis of sub-cellular differences in embryonic chick cardiomyocyte adhesion and alignment to the biomimetic and 20 × 20 fibronectin micropatterns as a function of cell density. (**A**) At low density, chick cardiomyocytes adhere and align directly to the 20 × 20 fibronectin lines due to spatial confinement and the inability to bridge across lines. (**B**) At high density, chick cardiomyocytes are able to bridge the 20 × 20 lines to form a confluent monolayer that is still aligned. (**C**) At low density, chick cardiomyocytes adhere to the various features in the biomimetic pattern and are not well aligned. (**D**) At high density, chick cardiomyocytes adhere to the entire surface of the biomimetic pattern and are well aligned. (**E**) Heat maps of cell substrate coverage, showing the normalized occurrence of cardiomyocytes adhering to the biomimetic and 20 × 20 micropatterns at both low and high cell densities. (**F**) Histogram of substrate coverage for chick cardiomyocytes on the biomimetic pattern at low and high densities, indicating the normalized occurrence of a cell adhering to the biomimetic pattern, binned in to 1 µm^2^ regions. (**G**) Heat maps of cell OOP, showing regional variation in cardiomyocyte alignment on the biomimetic and 20 × 20 micropatterns at both low and high cell densities. (**H**) Histogram of OOP for chick cardiomyocytes on the biomimetic pattern at low and high densities. Scale bars are 20 µm. Heat maps and orientation plots were generated with a custom MATLAB code (MATLAB 2017a, https://www.mathworks.com/products/new_products/release2017a.html).

To better understand why cardiomyocytes on the biomimetic micropattern have this unique density-dependent behavior, we analyzed adhesion and alignment on specific sub-regions of the fibronectin micropattern. On the 20 × 20 control micropattern, cardiomyocytes at low density adhered exclusively to individual fibronectin lines and were highly aligned (Fig. 3A). At high density cardiomyocytes bridged between the fibronectin lines and maintained the high degree of alignment (Fig. 3B). In contrast, on the biomimetic micropattern, cardiomyocytes at low density adhered to multiple fibronectin features adopting a more spread morphology with lower alignment (Fig. 3C). Cell shape depended on the underlying fibronectin features, but in all cases they were able to bridge across multiple features. At high density the behavior changed, with cardiomyocytes spread over all features and myofibrils highly aligned in the pattern direction (Fig. 3D), as already shown at lower magnification (Fig. 2).

Next, we used quantitative image analysis to understand the role of cell-substrate interactions on cardiomyocyte alignment and how this changed between low and high cell density. We micropatterned substrates using fluorescently-labeled fibronectin and imaged location-specific actin orientation to create maps of cell adhesion and alignment. Unit cells for the biomimetic pattern (155 × 250 µm) and the 20 × 20 pattern (40 × 1 µm) were defined and then split into 1 × 1 µm bins. For each bin we calculated the percent substrate coverage based on detected actin filaments (Fig. 3E,F), OOP (Fig. 3G,H), and the mean, median, mode, and standard deviation of the actin orientation angles (Supplementary Fig. S5). The heat maps revealed that the biomimetic micropattern had areas of preferred adhesion at both low and high density, with specific features where cells were found nearly 100% of the time (Fig. 3E). Interestingly, while there were areas of the biomimetic micropattern that had high OOP, these only partially corresponded to areas of high adhesion (Fig. 3G). Cardiomyocyte adhesion to the most and the least populated areas of the biomimetic micropattern was 38:1 at low density and 2.6:1 at high density (Fig. 3E,F). This indicates that cell attachment and spreading was influenced much more by cell-substrate interactions at low density, when cell–cell interactions are uncommon. A similar effect was observed for cardiomyocyte OOP on both patterns (Fig. 3G,H), where region-specific variability at low density became a more uniform distribution at high density. These results suggest that at low density cardiomyocyte alignment is primarily driven by cell-substrate interactions, while at high density cell–cell interactions also contribute. Other heat maps quantifying cell alignment including mean, median, mode, and standard deviation of the orientation angle as well as their respective histograms further support this interpretation (Supplementary Fig. S5).

### iPS-derived cardiomyocytes do not show density-dependent alignment on the biomimetic micropattern

**Figure 4:**
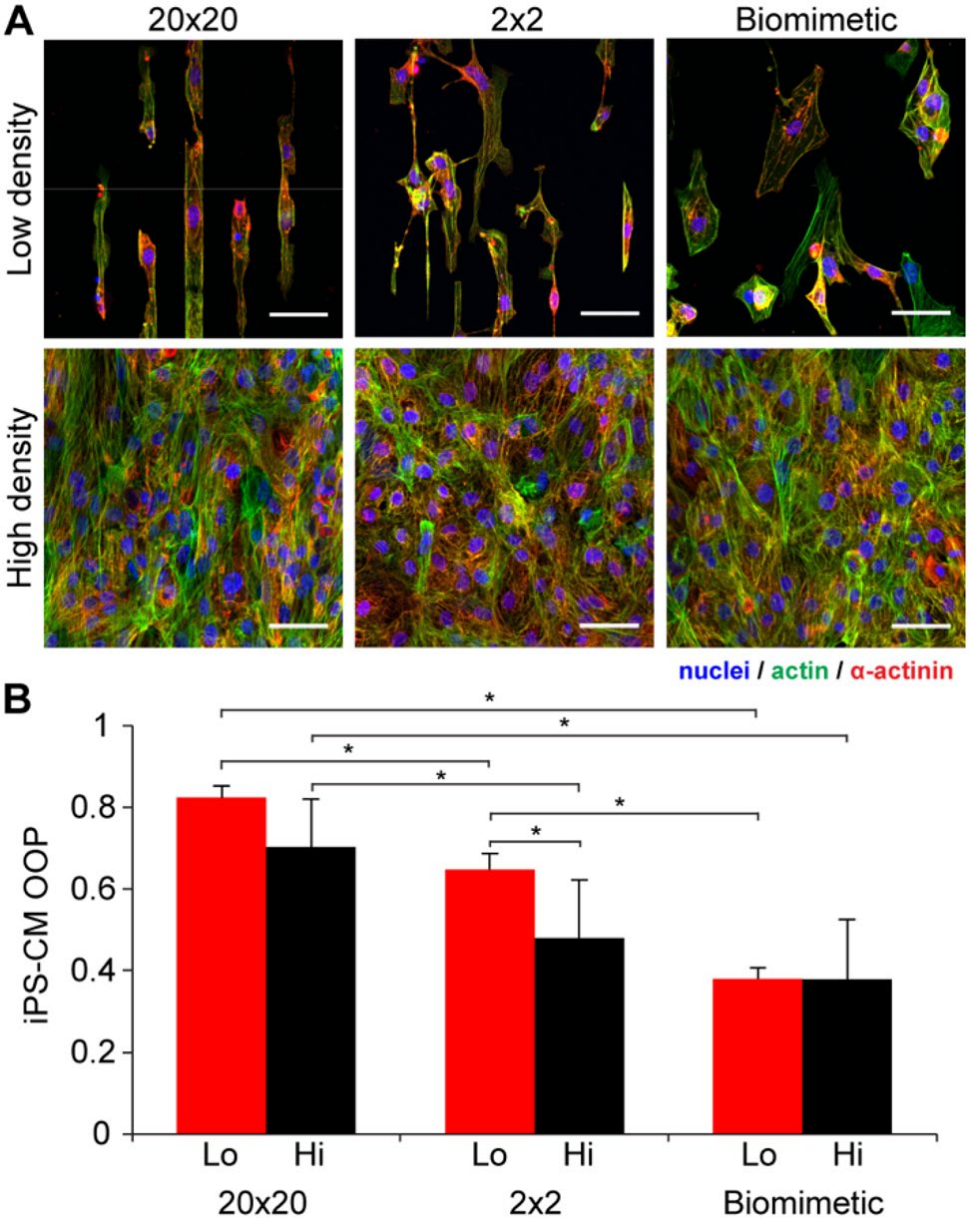
Analysis of iPS-CM adhesion and alignment on the fibronectin micropatterns at low and high cell density. (**A**) Representative images showing pattern-dependent difference in cell morphology at low density (isolated cells) and high density (monolayer). Scale bars are 50 µm. (**B**) OOP analysis of iPS-CMs at low and high cell densities showing that alignment is dependent on both density and pattern type. Note that results for the iPS-CMs are distinctly different than for the chick cardiomyocytes. Two-way ANOVA with Tukey’s posthoc, *indicates p < 0.05, n ≥ 3). Error bars are standard deviation.

A human-based model is required in order to determine if the behavior observed for embryonic chick cardiomyocytes holds in a more clinically relevant cell system. To create such a model, we used human induced pluripotent stem cell-derived cardiomyocytes (iPS-CMs) as a cell source for engineering cardiac monolayers on the fibronectin micropatterns, and differentiated them into cardiomyocytes using standardized protocols (Supplementary Figure, S6). The human iPS-CMs were seeded on the fibronectin micropatterns at low (60,000 cells/cm^2^) and high (250,000 cells/cm^2^) densities, cultured for three days, and then fixed, stained, and imaged using confocal microscopy. These iPS-CM seeding densities achieved comparable seeding of low (isolated cells) and high (monolayer) cell densities when compared to previous chick cardiomyocyte studies. Results showed that iPS-CMs have a more circular and less elongated shape compared to the chick cardiomyocytes on the same micropatterns (Fig. 4A). The actin OOP analysis revealed overall lower alignment of iPS-CMs compared to chick cardiomyocytes (Fig. 4B), and most notably a lack of density-dependent alignment on the biomimetic micropattern. Overall cardiomyocyte OOP was higher at low density for the line patterns (although only the 2 × 2 pattern showed statistically significant difference) and the same as the high-density OOP for the biomimetic pattern indicating that the influence of cell–cell and cell-substrate interactions on iPS-CM alignment is different compared to chick cardiomyocytes. This could also be caused by the immature phenotype of iPS-CMs^45^ as well as possible differences in the expression levels of fibronectin-specific integrins^46^.

### Cardiac fibroblasts combined with iPS-derived cardiomyocytes restore density-dependent alignment on biomimetic micropattern

The 2D engineered tissues produced using chick embryonic cardiomyocytes contain 49%±13% cardiomyocytes based on alpha-actinin expression, with other cell types being mainly fibroblasts, which decreases the cardiomyocyte OOP in a monolayer (Supplemental Fig. S4). The iPS-CMs, however, are obtained at a very high purity (88.5±10.6%) due to the lactate purification step of the differentiation protocol (Supplemental Figure, S6). Given the difference in behavior between the cardiomyocytes, we asked if there might also be a difference in the behavior of the fibroblasts between embryonic chick and human sources. Given that iPS-CMs are already known to be phenotypically immature^47^, we selected adult cardiac fibroblasts (CFBs) to see if this cell type might alter cell behavior.

**Figure 5:**
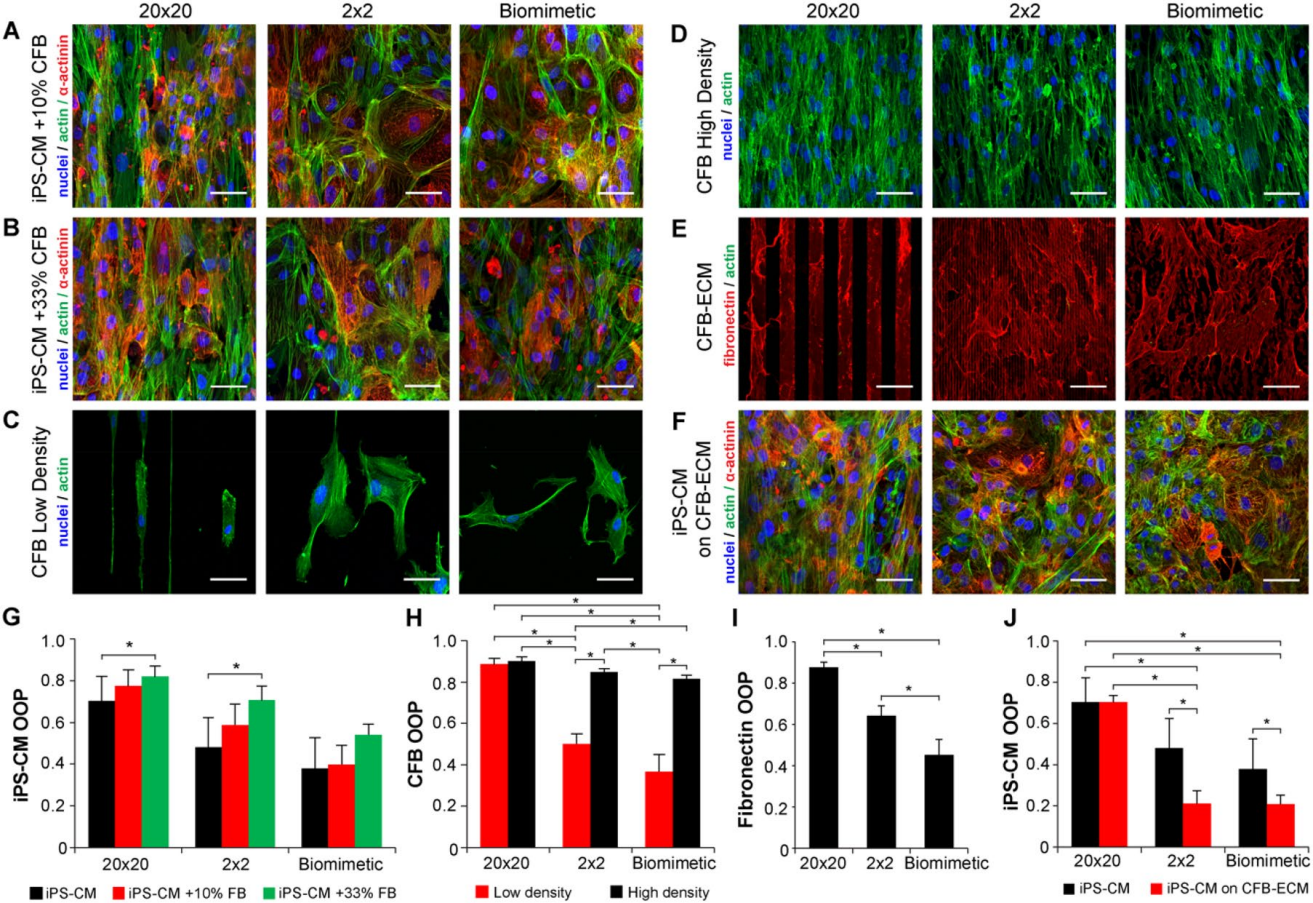
Assessing the role of CFBs on the alignment of iPS-CMs as a function of the fibronectin micropattern. (**A**) Representative images of iPS-CMs with 10% CFBs on the fibronectin micropatterns. CFBs can be distinguished from cardiomyocytes by the lack of α-actinin (red) staining. (**B**) Representative images of iPS-CMs with 33% CFBs on the fibronectin micropatterns. Note the increase in cell alignment relative to (**A**). (**C**) CFBs on the fibronectin micropatterns at low density show pattern-dependent alignment. (**D**) CFBs on the fibronectin micropatterns at high density show alignment on all patterns. (**E**) Fluorescent staining of fibronectin generated by CFBs cultured on the micropatterned substrates, after CFB removal. The 20 × 20 pattern has significantly less CFB-generated fibronectin, likely due to the weaker attachment to the original fibronectin pattern. (**F**) Representative images iPS-CMs cultured on the CFB-generated ECM shown in (**E**). (**G**) Alignment analysis of iPS-CMs on the fibronectin micropatterns with 0%, 10% or 33% addition of CFBs shows increased OOP with increased CFB percentage for all patterns. (**H**) Alignment analysis of CFBs on the fibronectin micropatterns shows that at low density CFB OOP is pattern-dependent, but at high density it is comparably high for all three patterns. (**I**) Alignment analysis of fibronectin generated by CFBs after CFB removal from the surface (CFB-ECM) reveals that for the 2 × 2 and biomimetic patterns, OOP for CFB-ECM is much lower than for the CFB actin cytoskeleton. (**J**) Alignment analysis of iPS-CMs cultured on the CFB-ECM shows decreased OOP for the 2 × 2 and biomimetic conditions compared to the iPS-CM controls cultured on the fibronectin micropatterns alone. Scale bars are 50 µm. (**H**), (**J**) Two-way ANOVA with Tukey post hoc, *indicates p < 0.05, n ≥ 3. (**G**), (**I**) One-way ANOVA with Tukey post hoc, *indicates p < 0.05, n ≥ 3

To test this, we mixed together primary human CFBs with the purified iPS-CMs to determine the effect on the alignment in monolayer culture. We added 10% (Fig. 5A) and 33% (Fig. 5B) CFBs to iPS-CMs and seeded them on the fibronectin 20 × 20, 2 × 2, and biomimetic micropatterns. Human CFBs and their respective concentration were chosen based on their previous use in the 3D engineered cardiac tissue literature^48,49^. After 3 days of incubation we observed that the iPS-CMs with CFBs appeared to have increased alignment compared to iPS-CM only condition. Quantitative analysis of OOP confirmed this with a trend toward increased alignment with CFB concentration for all three pattern types, and a statistically significant increase on the 20 × 20 and 2 × 2 micropatterns (Fig. 5G). Next, we asked if the CFBs by themselves had the ability to align to the micropatterns. We analyzed the CFB response to the fibronectin patterns at low (30,000 cells/cm^2^) and high (250,000 cells/cm^2^) densities. Results showed that at low density, alignment was high on the 20 × 20 micropattern but low on the 2 × 2 and biomimetic micropatterns, where the CFBs could easily spread across lines or features (Fig. 5C). In contrast, at high density the CFBs were highly aligned on all micropatterns (Fig. 5D), showing a similar density dependent alignment to that previously observed for the chick cardiomyocytes on the biomimetic micropattern (Fig. 2A,B). The OOP analysis confirmed that CFBs have lower alignment at low density and significantly higher alignment at high density on the 2 × 2 and biomimetic micropatterns (Fig. 5H). These results reveal that the iPS-CMs and CFBs behave differently than the chick cardiomyocytes and fibroblasts. Further, in the mixed iPS-CM and CFB cultures it appears that the CFBs are responsible for the density dependent alignment.

Finally, we sought to determine if the CFB-mediated alignment was caused by cell–cell interactions between CFBs and iPS-CMs, interactions between iPS-CMs and CFB-generated ECM, or a combination thereof. To assess this, we cultured only CFBs on the fibronectin micropatterns for 3 days to produce aligned and confluent monolayers (as seen in Fig. 5D). During this time the CFBs generated significant amounts of fibronectin-rich ECM. To determine if iPS-CM interaction with this CFB-generated ECM was responsible for the density dependent alignment, CFBs were lysed using 2M Urea solution and removed from the substrate. Staining the fibronectin in the CFB-generated ECM showed the formation of a dense fibrillar matrix and co-staining for F-actin confirmed that this decellularization process effectively removed cell components while preserving significant amounts of ECM (Fig. 5E). As the ECM had fibrillar structure, we were able to analyze its alignment and determine the OOP for the different micropatterns (Fig. 5I). Even though the actin alignment of the CFBs at high density was the same on all micropatterns (Fig. 5H), when looking at the fibronectin ECM only the 20 × 20 micropattern had this same high level of alignment. In comparison, the fibronectin ECM on the 2 × 2 micropattern had significantly lower alignment and the fibronectin ECM on the biomimetic patterns had even lower alignment. We next seeded iPS-CMs on the CFB-generated fibronectin ECM and observed high cell alignment on the 20 × 20 micropattern, but decreased alignment on the 2 × 2 and biomimetic micropatterns (Fig. 5F). Alignment analysis revealed that while the 20 × 20 pattern produced the same iPS-CM OOP with and without CFB-generated ECM, OOP was significantly lower on the 2 × 2 and the biomimetic patterns with CFB-generated ECM (Fig. 5J). These results demonstrate that the CFB-generated ECM is not responsible for the increase of OOP upon the addition of CFBs to iPS-CMs, suggesting that it is primarily cell–cell interactions that are responsible. Further confirmatory studies, including N-cadherin blocking experiments, were not pursued with iPS-CM monolayers because our previous chick studies demonstrated that N-cadherin blocking is ineffective at high cell densities.

### Induced maturation of iPS-derived cardiomyocytes improves alignment

**Figure 6:**
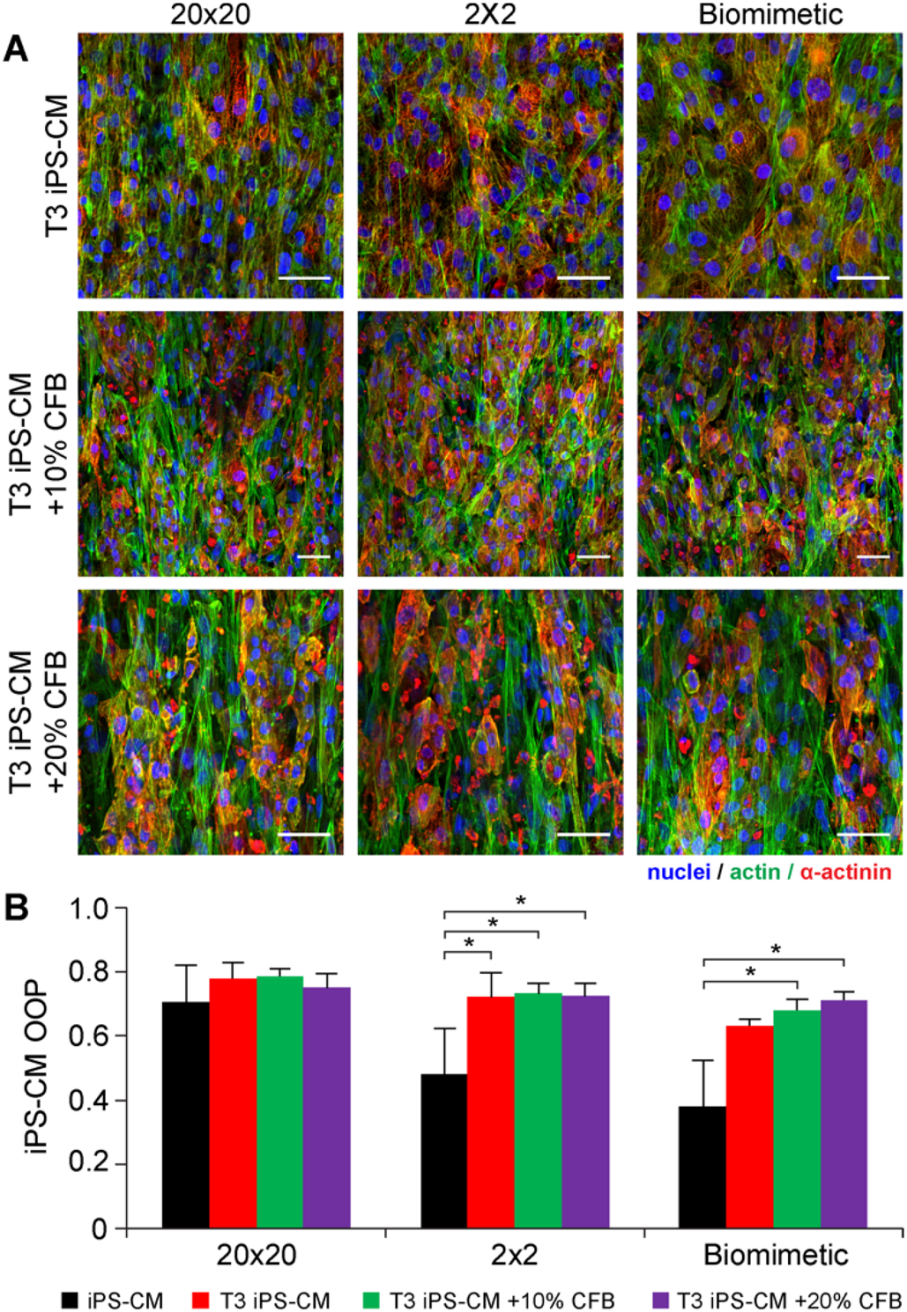
Understanding the relative role of CFB and T3 hormone on the alignment of iPS-CMs on the fibronectin micropatterns. (**A**) Representative images of iPS-CMs cultured on the fibronectin micropatterns with T3 hormone and with either 0%, 10%, or 20% CFBs. CFBs can be distinguished from cardiomyocytes by the lack of α-actinin (red) staining. Scale bars are 50 µm. (**B**) Alignment analysis of T3-matured iPS-CMs shows that T3-maturation significantly increases iPS-CM OOP on the 2 × 2 and biomimetic patterns. Interesting, the combination of CFBs and T3 hormone has no significant effect on alignment. One-way ANOVA with Tukey post hoc for each pattern, *indicates p < 0.05, n ≥ 3.

One of the main problems of using iPS-CMs for tissue engineering is the relative immaturity of these cells as characterized by multiple functional and structural characteristics, including shorter cell aspect ratio, fewer sarcomeres, poor sarcomere structure, and decreased contractile force^34,50–52^. We hypothesized that the immaturity of the iPS-CMs was responsible for the low actin alignment on the fibronectin micropatterns compared to chick embryonic cardiomyocytes, though no direct comparison of maturation state was performed between these two cell types. Although there is currently no known protocol to achieve an adult-like, or even later stage fetal-like phenotype without long-term electrical and mechanical stimulation^10,53^, several studies have shown that media formulations containing bioactive factors or certain metabolites can result in increased cardiomyocyte maturation^54–58^. In particular, incubation with T3 hormone (tri-iodo-L-thyronine) is one way to increase cell aspect ratio, number of sarcomeres, and contractile force of cardiomyocytes^56,59^. Here we asked if incubation of the iPS-CMs with the T3 hormone would cause a change in behavior in terms of alignment to the fibronectin micropatterns and make them respond more like the chick cardiomyocytes. The T3-matured iPS-CMs were seeded on the fibronectin micropatterns at high density to form a monolayer, either by themselves or mixed with CFBs at 10% or 20%, and incubated for 3 days. Immunofluorescent staining revealed that the T3-matured iPS-CMs were aligned on all micropatterns under all seeding conditions (Fig. 6A). Actin alignment analysis showed interesting results that depended on the specific micropatterns (Fig. 6B). For the 20 × 20 micropattern, iPS-CM alignment was high but the T3 treatment and the addition CFBs did not increase the OOP relative to the iPS-CM without T3 treatment. In contrast, on the 2 × 2 micropattern iPS-CM alignment significantly increased with T3 treatment and with the addition of CFBs, but there was no additional increase due to the CFBs. Finally, on the biomimetic micropatterns iPS-CM increased with T3 treatment but was not statistically significant. However, the T3 treatment combined with CFBs did produce a statistically significant increase in alignment. Taken together, these results suggest that the impact of T3 treatment and the addition of CFBs on iPS-CM alignment is highly dependent on the fibronectin micropattern. This indicates that there is a complex interaction between cell–cell interactions, cell matrix interactions, and maturation states, and this can change based solely on the geometry of the underlying micropattern.

## Discussion

It is well established that fibronectin plays a crucial role during embryonic development, including formation of the heart and coronary vasculature^36,60–62^. However, this process is still not completely understood and the mechanisms by which fibronectin regulates interactions between cells, other ECM components, and signaling molecules are still areas of active research^63–65^. Recent studies have shown that micropatterned fibronectin can control cardiomyocyte alignment^7,33,34,66^, which suggests that a similar process may occur in the developing heart. The biomimetic micropattern we created, inspired by the structure of fibronectin in the embryonic heart (Fig. 1), enabled us to investigate this possibility as well as elucidate factors important for cell alignment. Specifically, by determining the effect of cell density on chick cardiomyocyte alignment on various fibronectin patterns, we have shown that cytoskeletal orientation is primarily driven by cell-substrate interactions at low density, with an increasing role of cell–cell interactions at higher density (Figs. 2, 3). The reason this effect was observed so distinctly on the biomimetic micropattern, and not on the line micropatterns, lies within the relationship between the alignment induced by cell–cell interactions and that by cell-substrate interactions. Particularly, on the 20 × 20 and 2 × 2 lines, chick cardiomyocytes were highly aligned by these micropattern geometries at low density, so increasing the cell density had no additional affect (Fig. 3A). In contrast, on the biomimetic micropattern chick cardiomyocytes were able to spread laterally over multiple features at low density (Fig. 3C), which decreased alignment. In this case increasing cell density, and hence cell–cell interactions, significantly increased alignment. These results were supported by the N-cadherin blocking experiments, where within a range of intermediate cell densities this blocking resulted in significantly decreased alignment (Fig. 2G), even though only a fraction of cell–cell interactions were inhibited. As the biomimetic micropattern was based on the fibronectin structure of embryonic heart, we conclude that this fibronectin structure alone is not responsible for driving high myocardial alignment, and that cell–cell interactions known to transmit mechanical forces must play a pivotal role.

An important question is whether the density-dependent alignment on the biomimetic micropattern is due to cell–cell binding, physical packing, or a combination of the two. The N-cadherin blocking experiments suggest there is some role played by cell–cell adhesion molecules, and the effect was overridden at higher densities. This might be due to all N-cadherin not being blocked as the cardiomyocytes make more contact at higher cell densities, and enough N-cadherin remains available for binding. Cardiomyocytes also have other cell–cell adhesion molecules including desmosomes and gap junctions that are critical for electromechanical coupling^37,67,68^ and these interactions presumably also increase with cell density. However, physical packing is also a possible cause for the alignment. Though not widely reported for mammalian cells, dense populations of bacteria within confined spaces have been shown to self-organize due to biomechanical interactions^69^. Specifically, bacterial cells in part due to their elongated shape can form highly aligned multicellular structures^70,71^. Differential migration rates can also drive alignment^72^, and this may be a potential mechanism as well in the mixed cardiomyocyte and fibroblast cultures we used. Further, spatial confinement is known to be important in controlling assembly of the cytoskeleton^73^, and in fact actin filaments will self-assemble into aligned filaments under confinement even in cell-free systems^74^. Thus, biophysical mechanisms remain a possible driver of the density-dependent cell alignment.

Cell type also appears to be an important factor that impacts the degree that cells can align on the biomimetic micropattern. For example, chick fibroblast-rich populations showed lower alignment compared to cardiomyocyte-rich populations at the same seeding density (Supplemental Fig. S4). Independent analysis of cardiomyocyte and fibroblast alignment within the same culture revealed that at high density the alignment of cardiomyocytes and fibroblasts was the same, while at low density fibroblast alignment was lower than that of the cardiomyocytes (Supplemental Fig. S4). This shows an intrinsic difference in alignment between cell types, and it also shows that cardiomyocytes can directly influence the fibroblasts and improve their alignment in co-culture. While the mechanism of this is not known, it is likely some combination of physical, mechanical and biochemical cell–cell interactions. For the human iPS-CMs and CFBs, this response was essentially the opposite, with CFBs showing better alignment on the biomimetic micropatterns than the iPS-CMs. Importantly, adding the CFBs to the iPS-CMs at various concentrations showed a dose-dependent increase in alignment. This is similar to other studies that have shown that fibroblasts in 3D culture can facilitate the formation of functional tissue by remodeling ECM and creating favorable environment for promoting cardiomyocyte maturation and organization^75–77^. This shows that there are differences in the way cells align, that this depends on both cell type and species, and that results cannot be simply extrapolated from one to the other. Finally, the incubation with T3 hormone to help drive maturation of the iPS-CMs shows that the state of the iPS-CM also plays a role in phenotypic behavior. It shows that, at a minimum, that iPS-CM response to engineered microenvironments like the biomimetic micropattern can be pharmacologically-manipulated. Indeed, given the emergence of iPS-CMs for heart regeneration but the realization that these cells are phenotypically immature, such maturation strategies either using compounds such as T3 hormone or combining with adult cells such as CFBs may both be ways to improve the formation of functional heart muscle.

As with any in vitro model, there are several limitations to the studies described in this paper. First, the goal of this study was to gain a mechanistic understanding of how cardiomyocytes align with respect to cell–cell and cell-ECM interactions, as well as biochemical factors. However, it should be stressed that this 2D environment is a simplified model when compared to the 3D environment normally present within heart muscle^78,79^. Also, an evaluation of cardiomyocyte maturation gene expression markers was not performed. Further, though we performed extensive alignment analysis, we did not perform cardiac tissue functional assays, such as contractility and calcium transient analysis. Given the dramatic structural differences we observed between the patterns with respect to cell density, cell type, and biochemical factors, it will be important to quantify both gene expression and function in future studies.

## Methods

### Isolation, immunofluorescent staining, and imaging of embryonic chick hearts

Embryonic chick hearts were isolated and imaged based on previously published methods^36^. The stages of chick embryos (up to day 8 post fertilization) used in these studies are not considered live vertebrate animals by current NIH guidelines and the IACUC guidelines at Carnegie Mellon University, and therefore do not require institutional approval. All methods were carried out in accordance with relevant guidelines and regulations. Briefly, White Leghorn eggs were incubated at 37 °C with 50% humidity until day 6 and hearts were dissected, removing the atria, outflow track, and right ventricle. The left ventricles were then cut at the base in order to lay flat for high resolution imaging, fixed for 15 min in 4% formaldehyde, and incubated for 2 hrs at 37°C in PBS with 0.1% Triton X-100 and 5% goat serum. Ventricles were then immunofluorescently stained for fibronectin overnight at 4°C in PBS using mouse anti-chicken fibronectin (Sigma-Aldrich, #F6140) at a 1:100 dilution, in addition to 3:100 dilution of Alexa Fluor 633-conjugated phalloidin (ThermoFisher, #A22284) to stain F-actin. This was followed by secondary incubation with goat anti-mouse antibodies conjugated to Alexa Fluor 546 (ThermoFisher, #A11030) at a 1:100 dilution. After each staining step, the samples were washed in PBS for three times for 30 min. One drop of NucBlue (ThermoFisher, #R37606) per mL was added during the second washing step after secondary staining to stain the nuclei. 3D images were obtained using a high resolution 63x oil objective (NA =1.4) on a Zeiss LSM700 confocal microscope. We used the “refractive index correction” function of the Zeiss Zen 2010 software to correct spherical aberration using the ratio of the refractive indices of the medium of the sample (n=1.33 for PBS) and of the immersion medium of the objective (n′ =1.518 for oil), ${\text{R}} = \frac{{\text{n}}}{{{\text{n}}^{\prime } }} = 0.88$. We set the laser power and gain to increase through the depth of the myocardium to maintain appropriate signal intensity in each slice of the final 3D image stack.

### Generation of the biomimetic micropattern

To engineer an ECM protein micropattern that mimics the structure of fibronectin in the embryonic heart we isolated 6-day-old embryonic chick hearts (Fig. 1A), and then fixed, fluorescently stained and imaged the fibronectin ECM in the wall of the left ventricle (Fig. 1B). At this stage of development, the majority of the myocardium is trabeculated, with only a few layers of compacted and aligned cardiomyocytes in the ventricular wall. Images of compacted, ventricular myocardium were cropped to remove fibronectin corresponding to forming blood vessels or the adjacent epicardium (Fig. 1C), filtered to remove features <1 µm in size (Fig. 1D), and converted into 2D binary image (Fig. 1E). Finally, regions from multiple 2D binary images were combined together to construct a “unit cell” of the biomimetic pattern (Fig. 2F) that could then be arrayed in 2D to create a photomask that covers a 5x5 mm area (Fig. 1G). Established microcontact printing methods were then used to generate PDMS elastomeric stamps and transfer fibronectin to the surface of PDMS-coated coverslips (as illustrated in Fig. S1, Supporting Information). The fidelity of protein transfer during microcontact printing was validated using fluorescently-labeled fibronectin for the biomimetic (Fig. 1H), 20 × 20 (Fig. 1I) and 2 × 2 (Fig. 1J) micropatterns.

### Fabrication of PDMS stamps

PDMS stamps with three micropatterns (20 × 20, 2 × 2, and biomimetic) were used to pattern fibronectin onto substrates for cells. The process of PDMS stamp fabrication is based on the previously described technique (Supplementary Fig. S1)^80^. Briefly, photoresist SPR 220.3 was spin-coated onto a cover glass, placed under the photomask containing the desired pattern on a horizontal surface, and exposed to UV-light through the mask. The exposed parts of the photoresist were washed away using developer MF-319, the cover glass was washed in distilled water and dried with a nitrogen gun. Then PDMS was cast on top of the photoresist layer, cured at 65°C, and stamps were cut out of the PDMS layer.

### Substrate preparation

PDMS-coated coverslips were prepared using Sylgard 184 silicone elastomer according to the previously published procedures^81^. Briefly, Sylgard 184 base and curing agent were mixed with the mass ratio of 10:1 followed by the mixing and defoaming in a Thinky conditioning mixer. Coverslips were spin coated at 4000 RPM with a thin PDMS layer and then put in an oven at 65°C for 24 h in order to cure. Fibronectin patterns were microcontact printed onto the PDMS-coated coverslips according to a previously described technique with minor modifications (Supplementary Fig. S1). Briefly, PDMS stamps were cleaned by sonication in 50% ethanol for 45–60 min and dried using pressurized nitrogen. Then the patterned side of each stamp was incubated in 50 µg/mL solution of human plasma fibronectin (unlabeled or labeled with Alexa Fluor 546) for 60 min, washed in sterile water, and dried with a nitrogen gun. The coverslip was UV-Ozone treated for 15 min, and then the patterned side of the stamp was brought in contact with the coverslip for 5 min. The pattern transfer was verified for each stamp by confocal microscopy using fluorescently labeled fibronectin.

### Embryonic chick cardiomyocyte isolation

All chick cardiomyocytes used in experiments were isolated from 8-day-old chick embryos as previously described with minor modifications^82^. The stages of chick embryos (up to day 8 post fertilization) used in these studies are not considered live vertebrate animals by current NIH guidelines and the IACUC guidelines at Carnegie Mellon University, and therefore do not require institutional approval. All methods were carried out in accordance with relevant guidelines and regulations. First, the eggshells were opened, embryos removed, hearts cut out of the embryos and the atria removed leaving only ventricles. Then each ventricle was cut in 10–20 pieces and incubated in 1× TrypLE Express (Thermo Fisher) solution for 7 min at 37 °C. The supernatant was then removed and mixed with seeding medium (M199, 1% penicillin/streptomycin, 10% HI-FBS) for enzyme deactivation. New TrypLE Express solution was added to the minced hearts and the same procedure was repeated 4–6 times. After that, cell solution was centrifuged, resuspended in seeding medium, and pre-plated in T75 flasks 2 times for 45 min to remove fibroblasts from the solution. After pre-plating, cells were centrifuged and resuspended in seeding medium, cell density was counted, and cells were seeded onto the substrates fabricated above. After seeding, cells were kept in seeding medium for 24 h, then the medium was changed to maintenance medium (M199, 1% penicillin/streptomycin, 2% HI-FBS) in order to slow down fibroblast proliferation.

### Human iPSC culture and differentiation

Human iPSC line 13FLVNOC1 was provided by Prof. Joseph Wu’s lab in the Cardiovascular Research Institute at Stanford University. The iPSCs were cultured in Matrigel-coated 6-well plates in E8 medium and passaged using EDTA. The iPSCs were then differentiated into cardiomyocytes based on a previously described monolayer technique with minor modifications (Supplementary Fig. S6) ^83^. Briefly, on day 12 of differentiation, cardiomyocytes were passaged into Matrigel-coated wells and lactate purified in CDM3L medium (CDM3 medium without glucose supplemented with 5 mM sodium DL-lactate) for 7 days. After that cells were passaged into fibronectin-coated 6-well plates and cultured for an additional 7 days in CDM3L supplemented for the first 24 h with 10% fetal-bovine serum (FBS) and 2 µM thiazovivin. At 3 days prior to seeding on patterned substrates, CDM3L media was supplemented again with 10% FBS. After that cells were detached and seeded at the defined density onto the micropatterned PDMS-coated coverslips in CDM3 medium supplemented with 15% FBS and thiazovivin for the first 24 h and 10% FBS after that. For the experiments with matured cardiomyocytes, during the fibronectin conditioning stage iPS-CMs were cultured in CDM3 media supplemented with 20 ng/mL 3-iodo-L-thyronine according to the previously reported technique^59^.

### Fixation, staining, and fluorescent microscopy

Coverslips with cardiomyocytes and fibroblasts were fixed and permeabilized with 4% formaldehyde and 0.1% Triton-X 100 in PBS for 15 min and then washed 3 times with PBS. Coverslips were then stained with dilutions of 1:200 DAPI, 1:75 phalloidin conjugated to Alexa Fluor 488, and 1:200 primary antibodies, mouse anti-sarcomeric-α-actinin (Sigma A7811) or mouse anti-fibronectin (Sigma F3648). Samples were incubated with these stains for 60 min and then washed 3 times with PBS. Samples were then incubated in 1:100 dilution of goat anti-mouse secondary antibodies conjugated to Alexa Fluor 555 for 60 min and the washed 3 times in PBS. Finally, coverslips were mounted for imaging onto glass slides with ProLong preservative. All samples were imaged using confocal laser scanning microscopy (LSM 700, Carl Zeiss Microscopy, LLC).

### Cell alignment analysis

Cell actin alignment was measured using custom MATLAB code based on local actin filament orientation analysis based on adaptation of fingerprint analysis software^34,66,84^. To do this, confocal images of samples stained for nuclei, F-actin, and sarcomeric α-actinin were used. First, local orientations of actin filaments were detected across the image based on intensity gradients and then a filament mask was created to select corresponding to actin filaments (note that at the imaging resolution used this included actin filaments, stress fibers and myofibrils). Actin and α-actinin channels were then processed to produce binary masks of all cells (actin) and only cardiomyocytes (α-actinin). These masks were then used to determine cardiomyocyte actin orientation using the α-actinin mask, and fibroblast actin orientation by subtracting the α-actinin mask from the actin mask. Angular distribution of actin filaments was then used for each cell type to calculate the orientational order parameter (OOP), a measure of alignment varying between 0 and 1, where 0 corresponds to an isotropic distribution and 1 corresponds to a perfectly anisotropic distribution with all filaments aligned in the exact same direction. Heat maps and histograms were created by detecting location of the actin filaments relative to the fluorescently labeled fibronectin pattern and calculating alignment data for each location separately. Actin and α-actinin-based masks were also used to determine the cardiomyocyte purity and the overall cell surface coverage.

## Supplementary Information

Supplementary Information
