# Supplementary material for: Engineering aligned human cardiac muscle using developmentally inspired fibronectin micropatterns
Source: Sci Rep. 2021 Jun 1;11:11502. doi: 10.1038/s41598-021-87550-y (PMC8169656; doi:10.1038/s41598-021-87550-y)
Supplement: Supplementary file 1 — Supplementary Information [file 41598_2021_87550_MOESM1_ESM.docx]

Supplementary Information

Engineering Aligned Human Cardiac Muscle Using Developmentally Inspired Fibronectin Micropatterns

Ivan Batalov, Quentin Jallerat, Sean Kim, Jacqueline M. Bliley, and Adam W Feinberg*


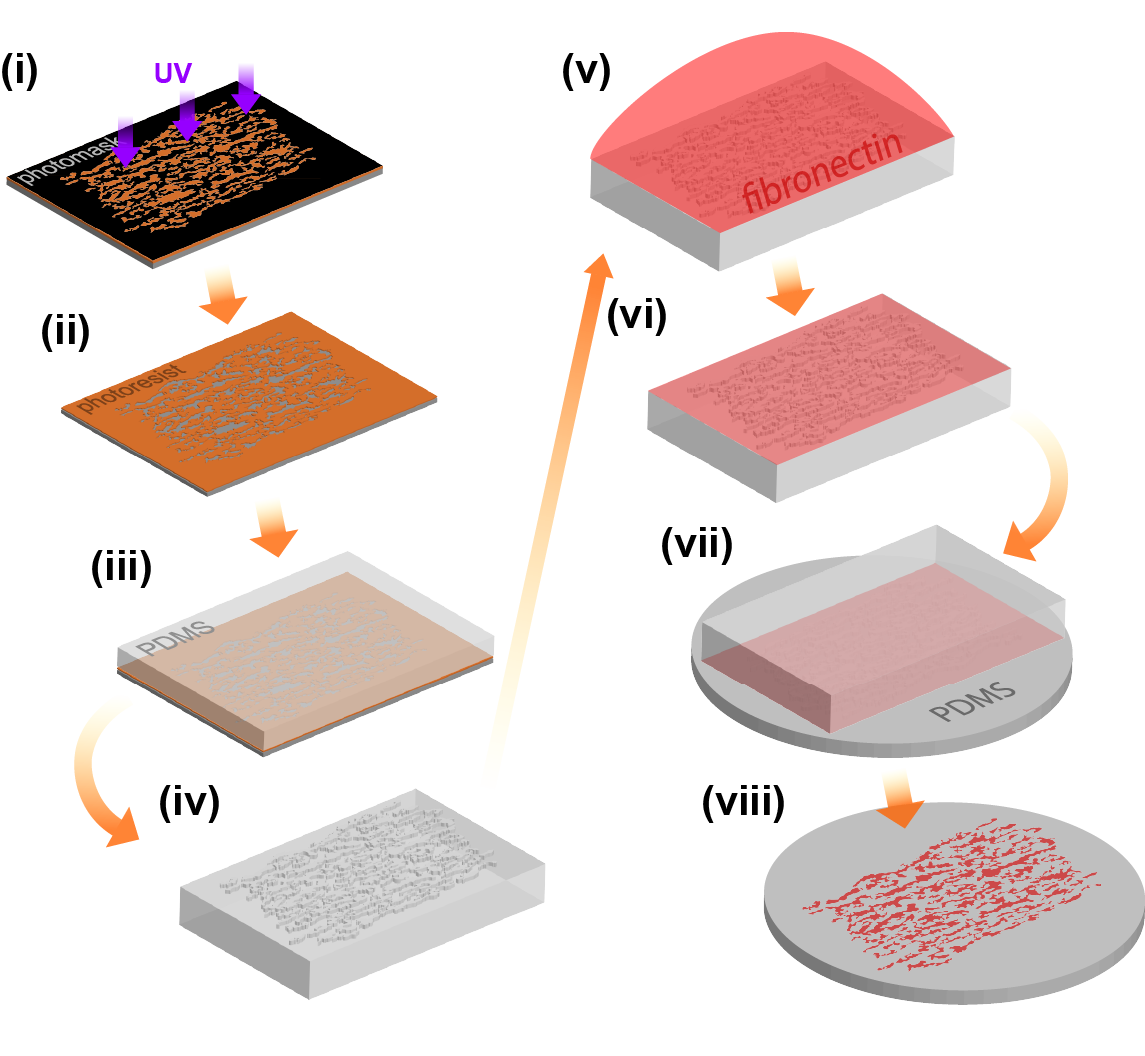


**Figure S1.** **Schematic of the process to microcontact print the ECM protein fibronectin onto a substrate.** (i) A layer of photoresist spincoated onto a coverslip is exposed to UV light through a photomask. (ii) The UV-exposed regions of the photoresist are removed through a developing step to create a master mold. (iii) PDMS pre-polymer is cast and cured on top of the master mold. (iv) The cured PDMS is peeled off the mold to create an elastomeric stamp. (v) The PDMS stamp is coated with fibronectin solution on the patterned side and incubated for 30 min. (vi) The PDMS stamp is washed and dried to create a layer of adsorbed fibronectin on the patterned side. (vii) The patterned side of the PDMS stamp is brought in to conformal contact with a UV-ozone pre-treated PDMS-coated coverslip. (viii) The PDMS stamp is removed, leaving a micropatterned layer of fibronectin on the PDMS-coated coverslip. Schematic was generated using Adobe Illustrator (2015, <https://www.adobe.com/products/illustrator.html>)


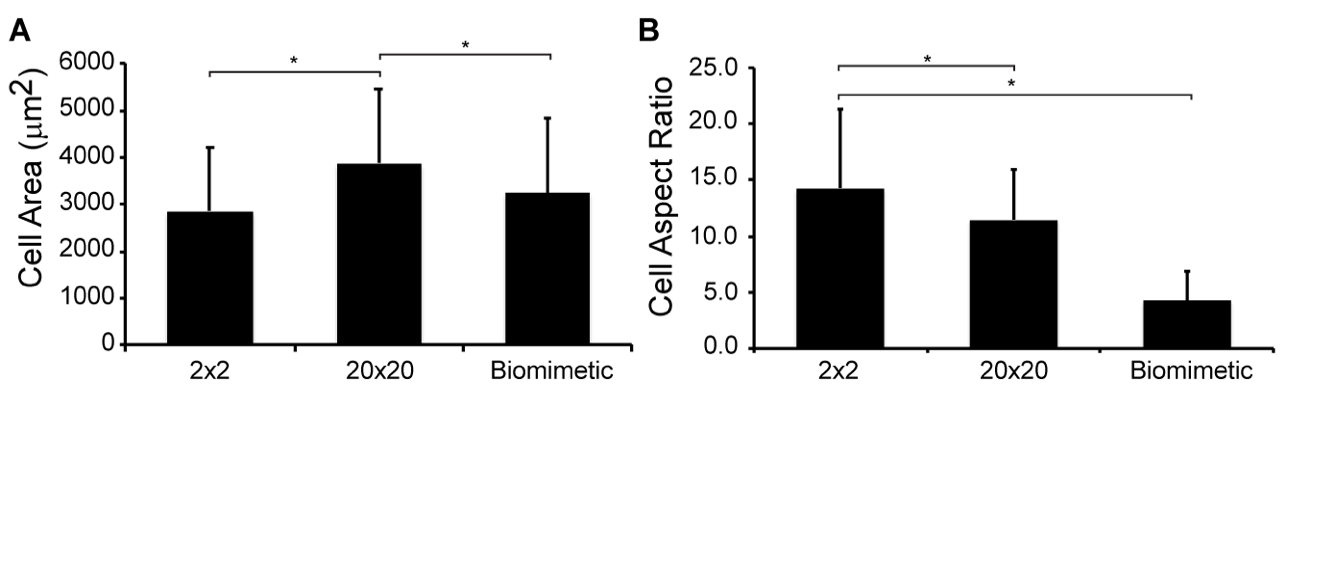


**Figure S2. Cell Area and Aspect Ratio on Chick Cardiomyocytes in Low Density Culture. (A)** Cell area on 2x2, biomimetic and 20x20 fibronectin micropatterns. **(B)** Cell aspect ratio on 2x2, biomimetic and 20x20 fibronectin micropatterns. Statistics based on one way ANOVA with a post hoc Tukey test. * indicates p<0.05.


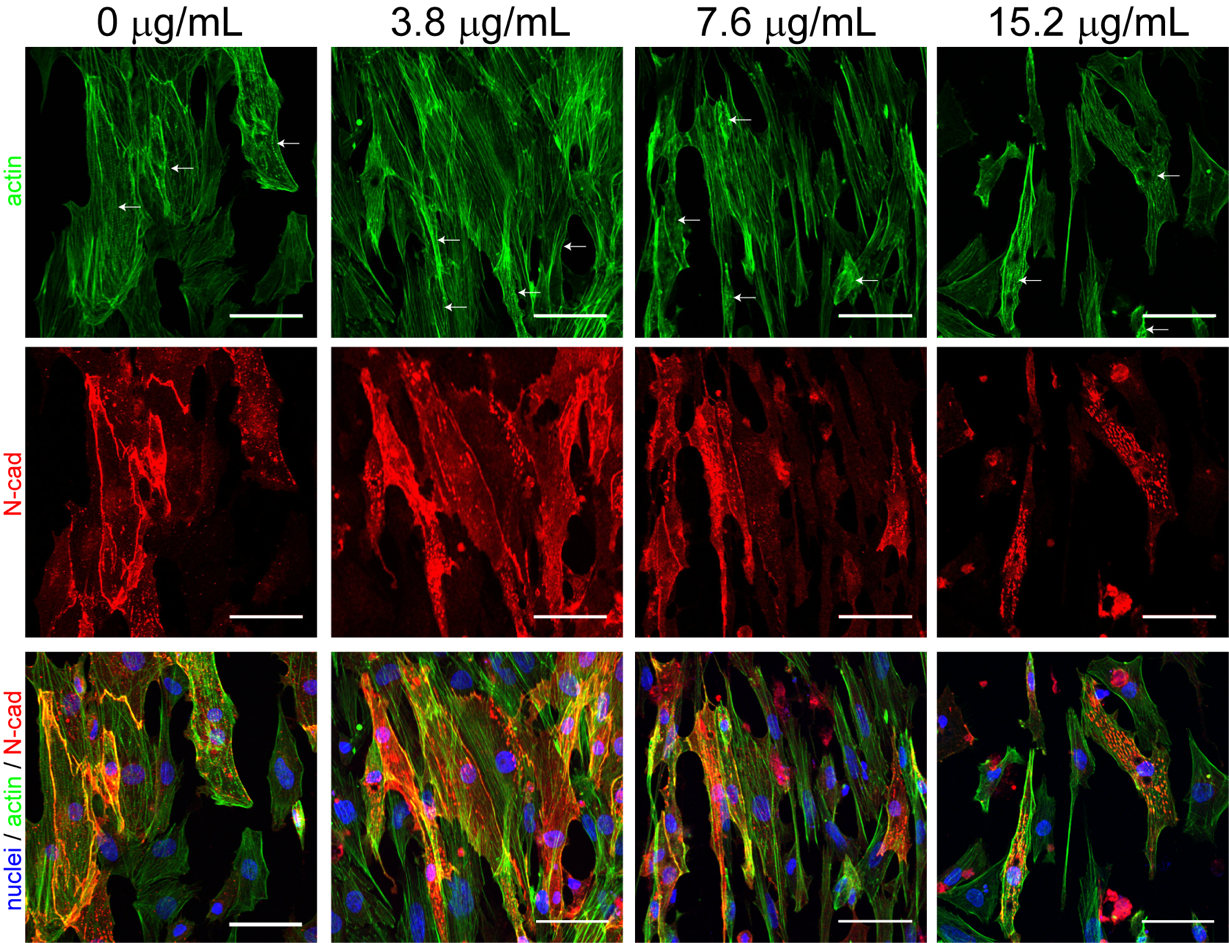


**Figure S3.** **N Cadherin Localization At Different Blocking Antibody Concentrations.** Analysis of N-cadherin localization in chick cardiomyocytes on biomimetic pattern as a function of increasing levels of incubations with the N-cadherin blocking antibody at 0, 3.8, 7.6 and 15.2 µg/mL. As the concentration of antibodies increases, N-cadherin staining is primarily internal and no longer at the cell-cell borders. Actin only channels are shown in top row, N-cadherin shown in middle row, and merged images are shown on the bottom row. White arrows indicate sarcomeric striations in actin channel, which were representative of cardiomyocytes. Scale bars are 20 mm.


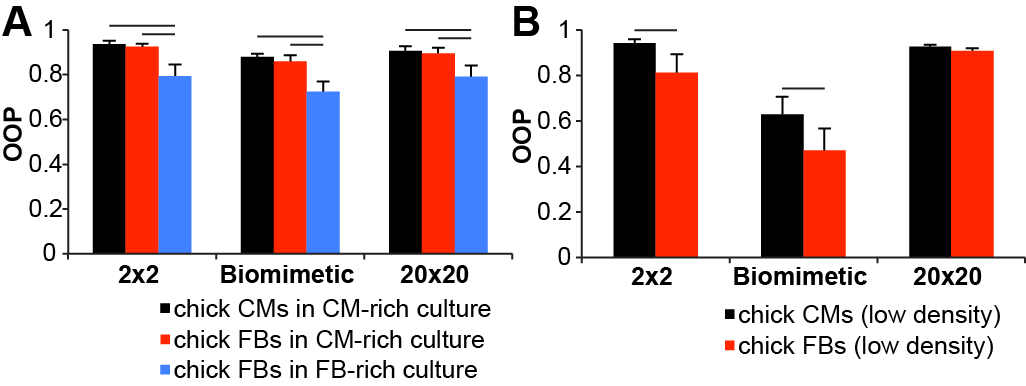


**Figure S4.** **Comparison of the alignment of both cardiomyocytes and fibroblasts (FBs) in the primary chick cardiomyocyte culture on the fibronectin micropatterns. (A)** Alignment analysis of cells seeded at high density comparing chick cardiomyocytes and FBs present in the same culture, as well as chick FBs alone. Note that in co-culture chick cardiomyocytes and FBs have equivalent OOP, but in culture alone chick FBs have significantly lower OOP on all patterns. **(B)** Alignment analysis of cells seeded at low density comparing chick cardiomyocytes and FBs present in the same culture. At low density cells do not contact one another, so separate FB only experiments were not required. Note that chick FB OOP is lower on the 2x2 and biomimetic patterns, but no difference was observed on the 20x20 pattern. Two-way ANOVA with Tukey post hoc, * indicates p<0.05, n≥3.


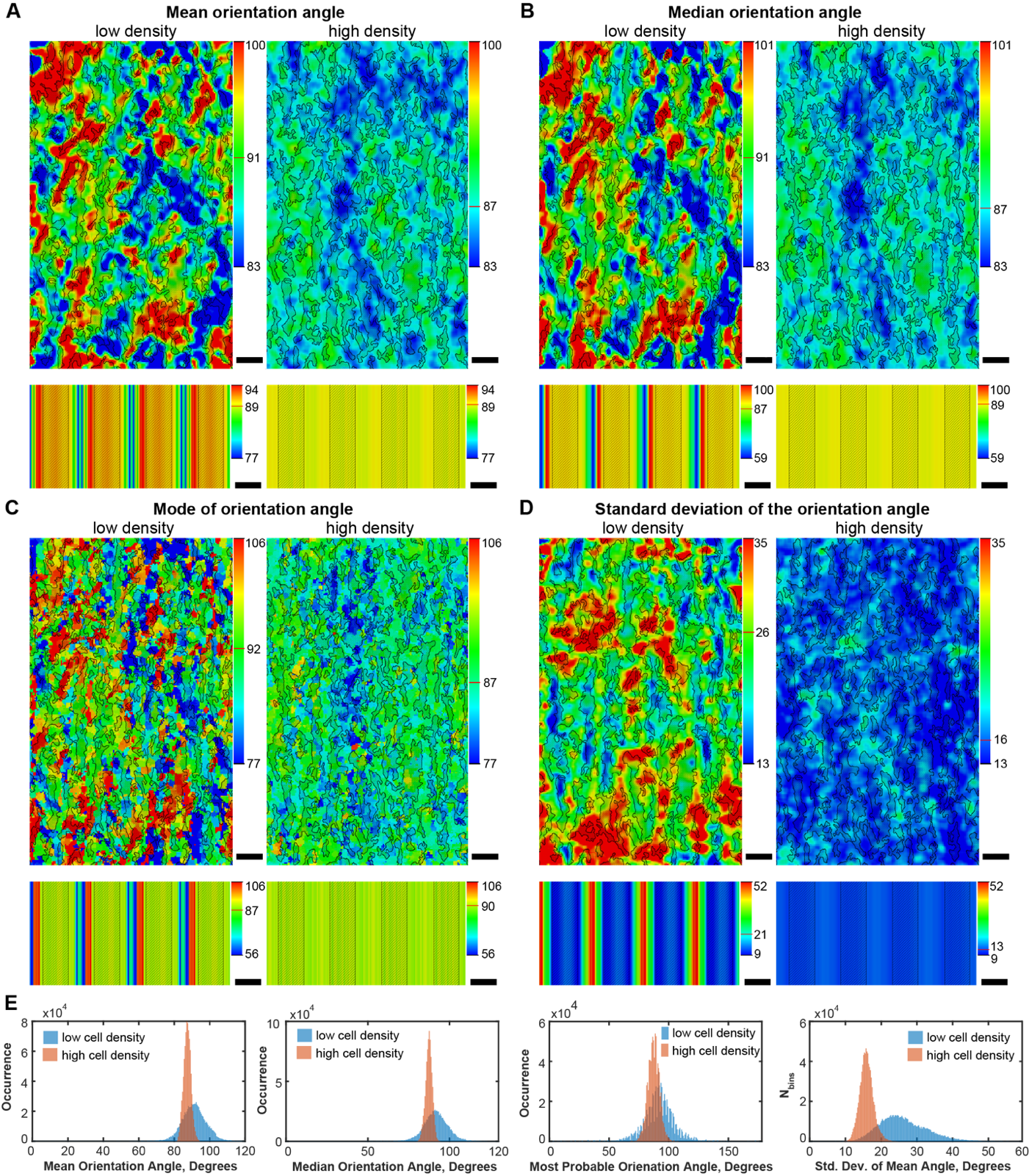


**Figure S5.** **Analysis of sub-cellular differences in embryonic chick cardiomyocyte angle of alignment to the biomimetic and 20x20 fibronectin micropatterns as a function of cell density.** **(A)** Heat maps of mean orientation angle on the biomimetic and the 20x20 patterns. **(B)** Heat maps of median orientation angle on the biomimetic and the 20x20 patterns. **(C)** Heat maps of the mode of the orientation angle. **(D)** Heat maps of standard deviation of the orientation angle on the biomimetic and the 20x20 patterns. **(E)** Histograms of mean, median, mode, and standard deviation of the orientation angle on the biomimetic pattern. Scale bars are 20 µm. Heat maps and orientation plots were generated with a custom MATLAB code (MATLAB 2017a, <https://www.mathworks.com/products/new_products/release2017a.html>).


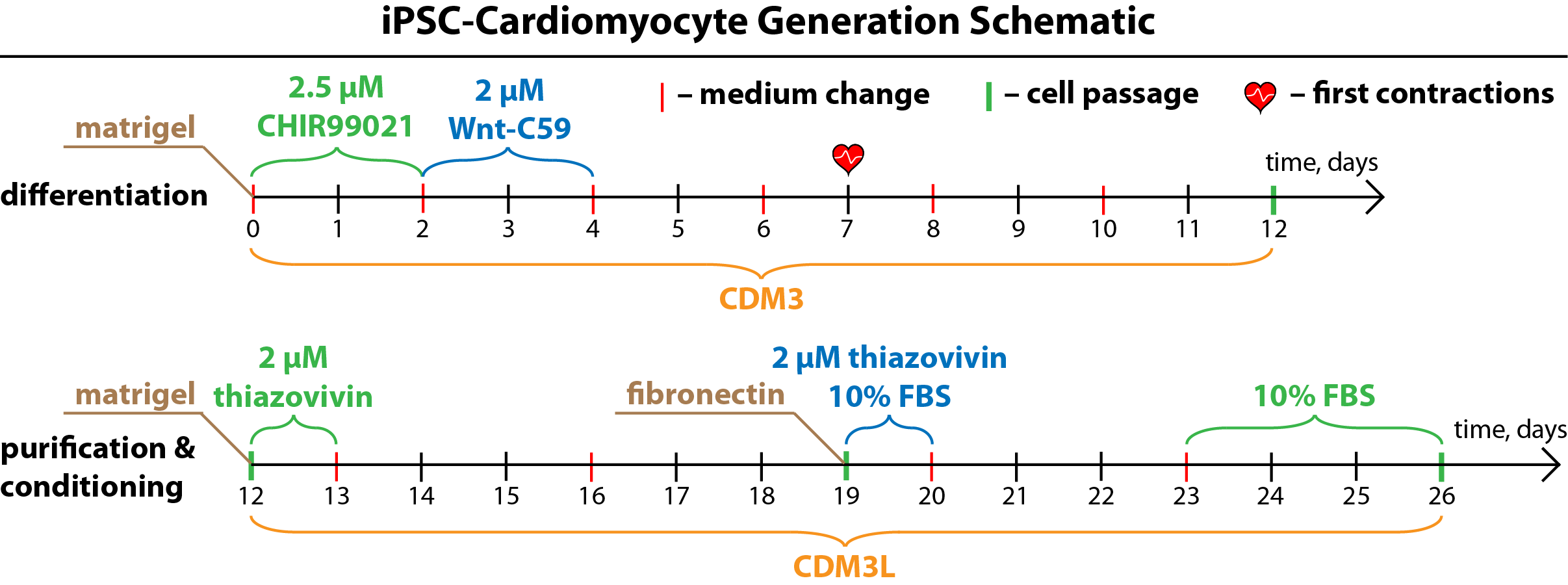


**Figure S6. Schematic of our modified process to differentiate iPS-CMs from the iPSC line 13FLVNOC1.** The iPS-CM generation has three stages. Stage 1 is a 12 day process consisting of Wnt-mediateddifferentiation from iPSCs in CDM3 media. Stage 2 is a 7 day process consisting of lactate purification to remove non-cardiomyocytes in CDM3L media. Stage 3 is a 7 day process consisting of conditioning of iPS-CMs on fibronectin in CDM3L transiently supplemented with 10% FBS to aid cell adhesion. The fibronectin conditioning is required to increase cardiomyocyte adhesion and spreading on the fibronectin micropatterns used in this study. Schematic was generated using Adobe Illustrator (2015).
